# Persistence rates of abatacept and TNF inhibitors used as first or second biologic DMARDs in the treatment of rheumatoid arthritis: 9 years of experience from the Rhumadata® clinical database and registry

**DOI:** 10.1186/s13075-019-1917-8

**Published:** 2019-06-06

**Authors:** Denis Choquette, Louis Bessette, Evo Alemao, Boulos Haraoui, Roelien Postema, Jean-Pierre Raynauld, Louis Coupal

**Affiliations:** 1 Rheumatology Research Institute of Montreal, Montréal, Canada; 2 Center for Osteoporosis and Rheumatology of Quebec (CORQ), Québec, Canada; 3 Bristol-Myers Squibb, Princeton, NJ USA; 4 Bristol-Myers Squibb, Uxbridge, UK

**Keywords:** Rheumatoid arthritis, Registry, Disease-modifying antirheumatic drugs (biologic), Persistence, Abatacept, TNF inhibitor

## Abstract

**Background:**

Treatment persistence is an important consideration when selecting a therapy for chronic conditions such as rheumatoid arthritis (RA). We assessed the long-term persistence of abatacept or a tumor necrosis factor inhibitor (TNFi) following (1) inadequate response to a conventional synthetic disease-modifying antirheumatic drug (first-line biologic agent) and (2) inadequate response to a first biologic DMARD (second-line biologic agent).

**Methods:**

Data were extracted from the Rhumadata® registry for patients with RA prescribed either abatacept or a TNFi (adalimumab, certolizumab, etanercept, golimumab, or infliximab) who met the study selection criteria. The primary outcome was persistence to abatacept and TNFi treatment, as first- or second-line biologics. Secondary outcomes included the proportion of patients discontinuing therapy, reasons for discontinuation, and predictors of discontinuation. Persistence was defined as the time from initiation to discontinuation of biologic therapy. Baseline characteristics were compared using descriptive statistics; cumulative persistence rates were estimated using Kaplan-Meier methods, compared using the log-rank test. Multivariate Cox proportional hazard models were used to compare the persistence between treatments, controlling for baseline covariates.

**Results:**

Overall, 705 patients met the selection criteria for first-line biologic agent initiation (abatacept, *n* = 92; TNFi, *n* = 613) and 317 patients met the criteria for second-line biologic agent initiation (abatacept, *n* = 105; TNFi, *n* = 212). There were no clinically significant differences in baseline characteristics between the treatments with either first- or second-line biologics. Persistence was similar between the first-line biologic treatments (*p* = 0.7406) but significantly higher for abatacept compared with TNFi as a second-line biologic (*p* = 0.0001). Mean (SD) times on first-line biologic abatacept and TNFi use were 4.53 (0.41) and 5.35 (0.20) years, and 4.80 (0.45) and 2.82 (0.24) years, respectively, as second-line biologic agents. The proportion of patients discontinuing abatacept and TNFi in first-line was 51.1% vs. 59.5% (*p* = 0.1404), respectively. In second-line, it was 57.1% vs. 74.1% (*p* = 0.0031). The main reasons for stopping both treatments were inefficacy and adverse events.

**Conclusions:**

Abatacept and TNFi use demonstrated similar persistence rates at 9 years as a first-line biologic agent. As a second-line biologic agent, abatacept had better persistence rates over a TNFi.

## Background

The goal of rheumatoid arthritis (RA) therapy, according to the Canadian Rheumatology Association Recommendations, is to achieve a target of sustained remission or low disease activity [1]. This should be achieved using a treat-to-target approach, based on shared decision-making between the patient and the rheumatologist regarding disease activity, and other patient factors, such as structural damage, comorbidities, and safety issues, as well as individual medical and societal costs [1, 2]. The current treatment strategy in Canada is to use a conventional synthetic disease-modifying antirheumatic drug (csDMARD), such as methotrexate (MTX), as a first-line treatment, in combination with either hydroxychloroquine or sulfasalazine and then add or switch to a biologic DMARD (bDMARD) or targeted-synthetic DMARD (tofacitinib) if the treatment target is not achieved. Tumor necrosis factor inhibitors (TNFis) are typically used as the first choice of biologic agent, although current guidelines do not stipulate any preference regarding which bDMARDs should be used [1, 2]. The Quebec general reimbursement policy states that reimbursement is available for the first biologic agent after the failure of two csDMARDs [3].

Approved treatments for the management of RA have multiple modes of action, and an understanding of how to use these treatments in clinical practice would benefit both clinicians and patients [2]. Several real-world studies have investigated the differences in the efficacy, after the first-line therapy had failed, between a strategy of cycling to another agent with the same mode of action and switching to an agent with a different mode of action [4–7]. Using a second-line TNFi after a first-line TNFi has failed can be an effective treatment strategy [8, 9], although second-line TNFi use often results in a lower response [4, 6, 7]. These results are supported by several studies reporting that switching to a different mode of action when first-line therapy fails is more effective than cycling between agents with the same mode of action [10–15]. Although these studies investigated the differences between cycling and switching in the real world, no long-term data are available to assess the long-term persistence rates between the two treatment strategies.

Abatacept, a selective T cell co-stimulation modulator [16], was one of the first approved alternatives to TNFi agents and, as such, has substantial effectiveness and safety data collected in both clinical trials and real-world practice, including information on the long-term follow-up of patients [17–24].

The aim of this analysis was to assess the long-term persistence of abatacept and a TNFi following an inadequate response to a csDMARD (first-line biologic agent initiation cohort) or a first bDMARD (second-line biologic agent initiation cohort), using data from patients with RA in Quebec, Canada, enrolled in the Rhumadata® registry.

## Methods

### Data source and patient population

The Rhumadata® registry is an observational clinical practice registry of approximately 7500 patients diagnosed with RA who fulfill the ACR criteria and were seen between January 1, 1999, and February 21, 2018, by 15 rheumatologists at either the Institute of Rheumatology of Montreal or the Center for Osteoporosis and Rheumatology of Quebec in Canada; 3471 patients are being actively followed. Patients included in Rhumadata® represent the standard referral to university or community rheumatology practice of the province of Québec.

Rhumadata® is a complete tabular electronic database built in MySQL language, an open source database provided by ORACLE® (Oracle Corporation, Redwood Shores, CA, USA). All fields are built as mandatory, limiting possible errors and variations of entry, with less than 5% of the information recorded as free text. A restricted verifiable personal usage code is allocated to all those who interact with the database. The data collected are saved twice daily on multiple secured servers. Data collected at baseline and/or at each visit are shown in Table 1.

**Table 1:** Summary of information collected in the Rhumadata® registry at baseline and/or each visit

Patient demographics	Age, gender, height, weight, date of appearance of first symptoms, date of diagnosis, and smoking status
Patient-reported outcomes	HAQ Disability Index, morning stiffness (minutes), pain (VAS), patient fatigue (VAS), and patient global evaluation of the impact of disease (VAS)
Physician-derived outcomes	PGA of disease activity (VAS), joint counts in 28 joints (tender joint count, swollen joint count), medications used for the control of the disease, and comorbidities and their pharmacologic treatment
Laboratory values	Complete blood count, ESR, CRP, liver function testing, creatinine level, RF, and anti-CCP (at baseline or once if not documented previously)
Safety information	AEs, SAEs, deaths, non-serious and serious infectious events, and antibiotic usage
Hospitalization	Surgeries, recent visits and length of stay in days to the emergency room or hospitalization at their local hospital

*AE* adverse event, *CCP* cyclic citrullinated peptide, *CRP* C-reactive protein, *ESR* erythrocyte sedimentation rate, *HAQ* Health Assessment Questionnaire, *PGA* physician global assessment, *RF* rheumatoid factor, *SAE* serious adverse event, *VAS* visual analog scale

The Rhumadata® registry was established in accordance with the Declaration of Helsinki and is approved on an annual basis by an ethics committee (IRB services); all patients provided written informed consent.

### Study population

This analysis includes all patients in the Rhumadata® database with a primary diagnosis of RA (based on the clinical judgment of the clinician) who were prescribed either abatacept or a TNFi (adalimumab, certolizumab, etanercept, golimumab, or infliximab) as a first or second biologic agent on or after January 1, 2006 (date of abatacept approval in Canada); all patients in the Rhumadata® registry meeting these selection criteria were included. Patients were followed from baseline, defined as the initiation of a first or second biologic agent, until the cessation of treatment, loss to follow-up, or the end of the analysis period (February 21, 2018), whichever comes first. Treatment assignment was based on clinical practice and determined by a rheumatologist.

### Study outcomes

The primary outcome was persistence to abatacept and TNFi treatments when used as first- or second-line biologic agents. Secondary outcomes included the proportion of patients discontinuing the treatment, reasons for treatment discontinuation, and predictors of treatment discontinuation.

Persistence was defined as the time on treatment and was calculated from initiation to discontinuation of biologic therapy; patients remaining on treatment at the time of data extraction and patients who were lost to follow-up were included in the analysis and were said to have a censored discontinuation time. Data from all patients who had temporary treatment interruption and subsequently resumed biologic treatment were also included in the analysis of the primary outcome, regardless of the length of the interruption. Time to treatment discontinuation was defined as the time taken until patients permanently stopped study treatments. Reasons for treatment discontinuation were recorded as well as secondary diagnoses and comorbidities reported at first or second biologic agent initiation and infections while on treatment.

### Statistical analysis

For baseline characteristics, data are presented as the number (%) for categorical variables and mean (SD) for continuous variables. Differences in categorical variables were tested using Fisher’s exact or chi-square tests and continuous variables using Student’s *t* test or ANOVA. DMARD persistence rates are presented using Kaplan-Meier survival curves, adjusted for censoring (i.e., for patients not experiencing biologic cessation during the study time frame for whatever reason) and compared using log-rank tests. These curves represent the attrition over time associated with drug persistence in the patient cohorts. A multivariate analysis was conducted using a subset of variables deemed to be univariately associated with DMARD persistence and/or a stepwise regression approach. Hazard ratios (HR) and 95% confidence intervals (CI) for time to treatment discontinuation were adjusted for age at diagnosis, disease duration, and age-adjusted Charlson Comorbidity Index.

Stepwise selection proportional hazard models (Cox regressions) were used to determine which, among all baseline variables measured, were associated with biologic discontinuation. Baseline characteristics potentially associated with DMARD persistence were included, one at a time, in proportional hazard models. Variables had to have a *p* value of 0.25 or less to enter the model and of 0.15 or less to remain in the model. The secondary diagnoses and comorbidities reported at first or second biologic agent initiation were tabulated for each treatment group, as were the infections reported while on treatment and the reasons for biologic discontinuation. Statistical analyses were performed using SAS version 9.4.

## Results

### Patient disposition and baseline characteristics

Overall, 705 patients were selected for first-line biologic agent initiation in this study following an inadequate response to a csDMARD; of these, 92 patients received abatacept and 613 received a TNFi. A total of 317 patients were selected for second-line biologic agent initiation, 105 of whom received abatacept and 212 of whom received a TNFi. No clinically significant differences were seen between the treatment groups for the majority of baseline characteristics, in both the first- and the second-line biologic agent initiation cohorts (Table 2); however, significant differences in a few key characteristics were noted (e.g., Clinical Disease Activity Index and Simplified Disease Activity Index scores and concomitant medications); these differences were controlled for using a multivariate analysis. In both first- and second-line biologic agent initiation cohorts, the majority of patients were female and received concomitant csDMARDs. In the first-line biologic agent initiation cohort, patients had a mean age of ~ 48 years and had been diagnosed with RA for ~ 7 years. In the second-line biologic agent initiation cohort, patients had a mean age at entry of 45 years and had been diagnosed with RA for 10–12 years.

**Table 2:** Baseline characteristics

	Use following csDMARD-IR			Use following first bDMARD-IR		
	Abatacept	TNFi	*p* value	Abatacept	TNFi	*p* value
*n*	92	613	–	105	212	–
Age, years	49.3 (13.6)	47.3 (13.2)	0.1871	45.9 (14.4)	45.5 (13.0)	0.8197
Disease duration, years	7.0 (7.8)	6.8 (7.7)	0.7980	12.1 (10.4)	10.0 (8.8)	0.0660
Female, *n* (%)	71 (77.2)	476 (77.7)	0.8940	84 (80.0)	156 (73.6)	0.2655
Concomitant medication use, *n* (%)						
csDMARDs	87 (94.6)	567 (92.5)	1.0000	87 (82.9)	174 (82.1)	1.0000
MTX	69 (75.0)	466 (76.0)	0.7955	69 (65.7)	142 (67.0)	0.8994
HCQ	72 (78.3)	350 (57.1)	< 0.0001	43 (41.0)	72 (34.0)	0.2640
SSZ	11 (12.0)	45 (7.3)	0.1455	9 (8.6)	10 (4.7)	0.2095
LEF	7 (7.6)	54 (8.8)	0.8434	7 (6.7)	16 (7.5)	1.0000
Corticosteroids	45 (48.9)	282 (46.0)	0.6542	73 (69.5)	93 (43.9)	< 0.0001
Number of oral corticosteroid prescriptions per 100 person-years of treatment	81.24	44.02		99.03	76.23	
TNFi used, *n* (%)	N/A			N/A		
Adalimumab		146 (23.8)			58 (27.4)	
Certolizumab		62 (10.1)			27 (12.7)	
Etanercept		239 (39.0)			69 (32.5)	
Golimumab		105 (17.1)			29 (13.7)	
Infliximab (Remicade)		60 (9.8)			26 (12.3)	
Infliximab (Inflectra)		1 (0.2)			3 (1.4)	
Comorbidities						
Age-adjusted CCI	2.8 (1.4)	2.4 (1.3)	0.2403	2.9 (1.8)	2.6 (1.5)	0.2228
Hyperlipidemia, *n* (%)	28 (30.4)	193 (31.5)	0.9044	42 (40.0)	75 (35.4)	0.4589
Hyperglycemia, *n* (%)	13 (14.1)	82 (13.4)	0.8699	11 (10.5)	32 (15.1)	0.2987
Hypertension, *n* (%)	37 (40.2)	290 (47.3)	0.2187	58 (55.2)	110 (51.9)	0.6329
COPD, *n* (%)	37 (40.2)	194 (31.6)	0.1211	43 (41.0)	76 (35.8)	0.3905
CVD, *n* (%)	11 (12.0)	75 (12.2)	1.0000	19 (18.1)	37 (17.5)	0.8769
Charlson Comorbidity Index	1.4 (0.9)	1.2 (0.7)	0.0039	1.4 (1.2)	1.3 (0.8)	0.59
RF+, *n* (%)	69 (75.0)	424 (69.2)	0.2680	73 (69.5)	141 (66.5)	0.7020
ACPA+, *n* (%)	56 (60.9)	358 (58.4)	1.0000	55 (52.4)	107 (50.5)	1.0000
ESR, mm/h	18.7 (16.0)	24.5 (19.8)	0.0080	21.9 (19.2)	24.8 (20.8)	0.3164
CRP, mg/L	14.30 (20.20)	12.90 (20.40)	0.5875	16.30 (23.40)	11.80 (19.40)	0.1226
Patient-reported outcomes						
Patient global, VAS 0–10	5.6 (2.4)	4.8 (2.8)	0.0430	5.2 (2.7)	4.4 (2.9)	0.0324
Patient pain, VAS 0–10	6.1 (2.6)	5.3 (3.0)	0.0401	5.7 (3.0)	5.0 (3.1)	0.1157
Patient fatigue, VAS 0–10	5.8 (2.8)	4.7 (3.2)	0.0099	5.7 (2.8)	4.8 (3.3)	0.0597
Morning stiffness, min	155.0 (308.3)	120.9 (274.1)	0.3676	127.3 (294.7)	93.0 (244.5)	0.3597
HAQ	1.31 (0.61)	1.24 (0.60)	0.3661	1.51 (0.60)	1.18 (0.66)	0.0006
Physician global, VAS 0–10	4.8 (2.7)	4.0 (2.6)	0.0370	4.0 (2.6)	3.4 (2.6)	0.2256
Swollen joint count, 0–28	8.5 (5.2)	7.4 (5.3)	0.1409	7.7 (5.3)	6.5 (6.0)	0.1755
Tender joint count, 0–28	6.8 (6.4)	6.6 (5.7)	0.8451	7.3 (6.1)	5.4 (5.9)	0.0481
Disease activity measures						
CDAI	26.3 (12.7)	23.5 (11.7)	0.1416	24.8 (11.0)	18.8 (12.5)	0.0069
SDAI	28.6 (13.1)	24.7 (12.0)	0.0639	26.3 (11.7)	20.4 (12.6)	0.0140
DAS28-4, ESR	4.8 (1.3)	4.7 (1.3)	0.7557	4.8 (1.2)	4.5 (1.4)	0.3621

Data are mean (SD), unless stated otherwise

*ACPA* anti-citrullinated protein antibody, *bDMARD* biologic disease-modifying antirheumatic drug, *CCI* Charlson Comorbidity Index, *CDAI* Clinical Disease Activity Index, *COPD* chronic obstructive pulmonary disease, *CRP* C-reactive protein, *csDMARD* conventional synthetic disease-modifying antirheumatic drug, *CVD* cardiovascular disease, *DAS28-4* disease activity score in 28 joints (four variables), *ESR* erythrocyte sedimentation rate, *HAQ* Health Assessment Questionnaire, *HCQ* hydroxychloroquine, *IR* inadequate response, *LEF* leflunomide, *MTX* methotrexate, *RF* rheumatoid factor, *SDAI* Simplified Disease Activity Index, *SSZ* sulfasalazine, *TNFi* tumor necrosis factor inhibitor, *VAS* visual analog scale

### Primary outcome

**Fig. 1:**
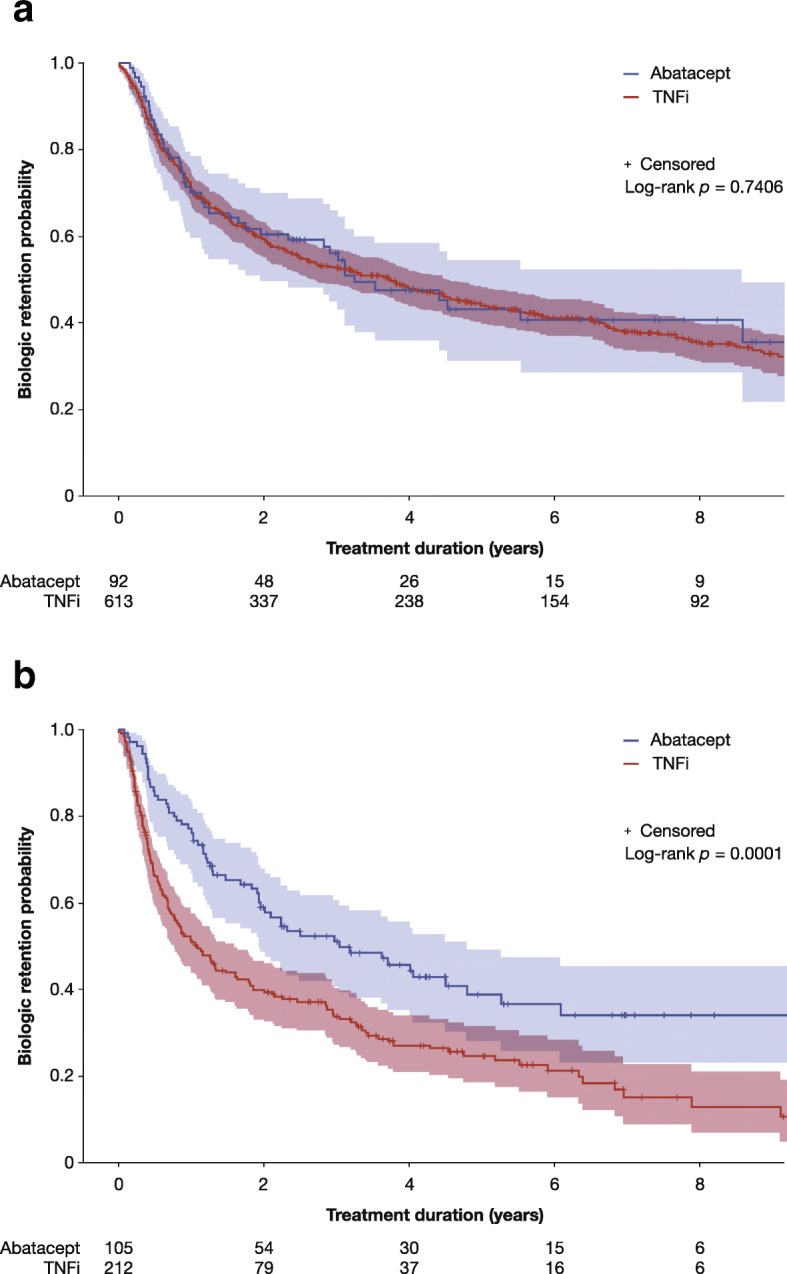
Kaplan-Meier retention curves in patients failing **a** csDMARDs and **b** a first bDMARD. bDMARD, biologic disease-modifying antirheumatic drug; csDMARD, conventional synthetic disease-modifying antirheumatic drug; TNFi, tumor necrosis factor inhibitor

In patients with an inadequate response to a prior csDMARD (first-line biologic agent initiation), there were no significant differences in the primary outcome of persistence during the 9-year study period between abatacept- and TNFi-treated patients (*p* = 0.7406; Fig. 1a); however, persistence was significantly higher with abatacept than with TNFi in patients who had an inadequate response to a prior bDMARD (second-line biologic agent initiation, *p* = 0.0001) (Fig. 1b). The mean (SD) biologic persistence times for the first-line biologic agent initiation were 4.53 (0.41) (median [95% CI], 3.25 [1.95, 8.59]) and 5.35 (0.20) (median [95% CI], 3.72 [2.71, 4.51]) years for the abatacept and TNFi groups, respectively. For the second-line biologic agent initiation, these values were 4.80 (0.45) (median [95% CI], 3.03 [1.92, 4.78]) and 2.82 (0.24) (median [95% CI], 1.08 [0.77, 1.60]) years, respectively. The mean (SD) treatment interruption time was 44.4 (133.1) days and 20.6 (64.6) days for first- and second-line TNFi use, respectively. For abatacept, the mean treatment interruption time was 34.7 (84.3) days and 37.4 (84.2) days for first-and second-line treatment, respectively.

Over the 9-year study period, a similar percentage of patients with an inadequate response to a prior csDMARD permanently discontinued both abatacept and a TNFi (51.1% vs. 59.5%, *p* = 0.1404); however, treatment discontinuation was significantly lower for abatacept than for TNFis in patients who had failed a first bDMARD (57.1% vs. 74.1%, *p* = 0.0031) (Table 3).

**Table 3:** Treatment status

Treatment status	Use following csDMARD-IR (first-line biologic agent)			Use following first bDMARD-IR (second-line biologic agent)		
	Abatacept	TNFi	*p* value	Abatacept	TNFi	*p* value
*n*	92	613	–	105	212	–
Stopped treatment, *n* (%)	47 (51.1)	365 (59.5)	0.1404	60 (57.1)	157 (74.1)	0.0031
Treatment duration, years, mean (SD)	1.57 (1.68)	2.01 (2.23)	0.1068	1.76 (1.78)	1.33 (1.70)	0.1045
Reasons for stopping, *n* (%)			0.1351			0.4409
Inefficacy	28 (59.6)	171 (46.8)		38 (63.3)	86 (54.8)	
Adverse event	8 (17.0)	76 (20.8)		12 (20.0)	27 (17.2)	
Lost to follow-up	2 (4.3)	21 (5.8)		0	6 (3.8)	
Treatment stopped by the patient	3 (6.4)	14 (3.8)		0	4 (2.5)	
Infections	3 (6.4)	22 (6.0)		2 (3.3)	9 (5.7)	
Death	0	7 (1.9)		3 (5.0)	4 (2.5)	
Ongoing treatment	45 (48.9)	248 (40.5)	0.1404	45 (42.9)	55 (25.9)	0.0031
Treatment duration, years, mean (SD)	4.46 (2.99)	6.25 (3.25)	0.0007	4.57 (2.69)	4.15 (2.68)	0.4364

*bDMARD* biologic disease-modifying antirheumatic drug, *csDMARD* conventional synthetic disease-modifying antirheumatic drug, *IR* inadequate response, *TNFi* tumor necrosis factor inhibitor

### Secondary outcomes

In both first- and second-line biologic agent initiation cohorts, the main reasons for stopping both abatacept and TNFi treatments were inefficacy (first-line, 59.6% vs. 46.8% [*p =* 0.1210]; second-line, 63.3% vs. 54.8% [*p =* 0.2851]) and adverse events (AEs) (first-line, 17.0% vs. 20.8% [*p =* 0.7006]; second-line, 20.0% vs. 17.2% [*p =* 0.7544]) (Table 3). In the first-line biologic agent initiation cohort, the mean (SD) time to treatment discontinuation was 1.57 (1.68) years for abatacept-treated and 2.01 (2.23) years for TNFi-treated (adjusted HR [95% CI], 0.932 [0.678, 1.252]) patients. In the second-line biologic agent initiation cohort, the mean (SD) time to treatment discontinuation was 1.76 (1.78) years for abatacept-treated and 1.33 (1.70) years for TNFi-treated (adjusted HR, 0.553; 95% CI, 0.403, 0.746) patients.

**Fig. 2:**
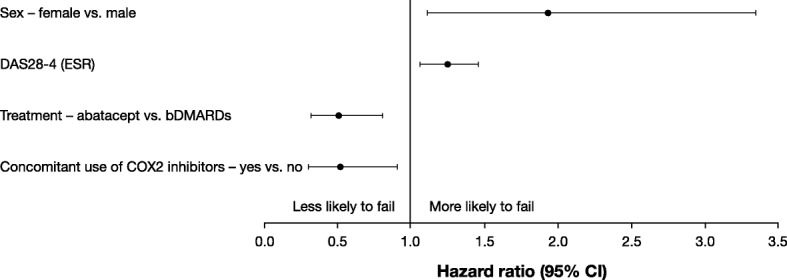
Multivariate analysis showing the predictors of biologic failure when used in patients with an inadequate response to a first bDMARD (second-line biologic initiation cohort). CI, confidence interval; COX2, cyclooxygenase 2; DAS28-4, disease activity score in 28 joints (four variables); ESR, erythrocyte sedimentation rate

Multivariate analysis showed that in patients with an inadequate response to a first bDMARD (second-line biologic initiation cohort), treatment with abatacept (vs. TNFi) (HR [95% CI], 0.506 [0.319, 0.804], *p* = 0.0039) and concomitant treatment with a cyclooxygenase 2 inhibitor (HR [95% CI], 0.524 [0.305, 0.901], *p* = 0.0194) were significant predictors of improved retention, whereas female (vs. male) sex (HR [95% CI], 1.933 [1.117, 3.345], *p* = 0.0185) and baseline disease activity score in 28 joints (four variables) (erythrocyte sedimentation rate) (DAS28-4 [ESR]) (HR [95% CI], 1.244 [1.064, 1.454], *p* = 0.0061) were significant predictors of biologic treatment failure (Fig. 2).

## Discussion

The results from this analysis of the Rhumadata® registry, using real-world data from all patients with a primary diagnosis of RA who were prescribed either abatacept or a TNFi as a first or second biologic agent in Canadian clinical practice, demonstrate that there was no difference in persistence during the 9-year study period between abatacept and a TNFi when used as a first-line biologic agent. As a second-line biologic agent, patients treated with abatacept had a greater persistence than those treated with a TNFi, based on both univariate (Kaplan-Meier) and multivariate (controlling for all baseline covariates using Cox proportional hazard model) analyses.

These findings are consistent with clinical trial data from the phase III AMPLE (NCT00929864) and ATTEST (NCT00095147) trials [22–24]. In the AMPLE trial, subcutaneous (SC) abatacept + MTX was non-inferior to SC adalimumab + MTX in terms of American College of Rheumatology 20% improvement (ACR20) response at 1 year, and the treatments remained comparable over the 2-year follow-up [23, 24]. Similarly, the ATTEST trial showed that ACR20 response rates were significantly greater with both abatacept + MTX and infliximab + MTX than with placebo + MTX at 6 months [22]. In both trials, similar safety profiles were shown between agents, although abatacept-treated patients had fewer serious adverse events and serious infections [22–24].

Registries complement data from randomized controlled trials, especially as more long-term experience, including persistence data, can be collected in real-world settings than is possible in clinical trials [25–27]. Several observational studies and biologic registries have contributed to a wealth of available data regarding the long-term efficacy and safety of these agents [18, 19, 21, 23, 25]. Furthermore, observational studies have highlighted the importance of real-world persistence, defined as the time from the index dose to the time of switching to a different biologic or the time of the last dose/censoring, as a surrogate for treatment effectiveness and a factor to consider when choosing between agents [20, 28]. These studies showed that first-line abatacept treatment had higher persistence rates than TNFis [20, 28].

Our results in patients treated with abatacept or a TNFi as a second-line biologic agent are supported by several real-world studies [12–14]. These studies showed that switching treatment to a biologic with a different mode of action after the first-line treatment (with TNFi) has failed is associated with better outcomes than cycling to a different TNFi [12–14]. A systematic review and Bayesian analysis comparing the effectiveness of switching to a non-TNFi vs. cycling between TNFi agents showed that switching to a therapy with a new mode of action was more effective than TNFi cycling in patients with RA and an inadequate response to an initial TNFi [29]. The cause of TNFi discontinuation may influence the performance of the cycling strategy as the differences were particularly evident in patients who failed the previous treatment due to inefficacy [12–14]. Our results further support this growing body of evidence, showing that as a second-line biologic agent, abatacept was associated with greater persistence than a TNFi over the 9-year follow-up period. In addition to better effectiveness, switching to a new mode of action rather than cycling to a different TNFi has been associated with a reduction in overall economic burden and healthcare costs [11, 30].

In our multivariate analysis of patients with an inadequate response to a first bDMARD (second-line biologic initiation cohort), treatment with abatacept (vs. TNFi) and concomitant treatment with a cyclooxygenase 2 inhibitor were significant predictors of improved retention, whereas female (vs. male) sex and DAS28-4 (ESR), measured continuously, were significant predictors of treatment failure. The published literature looking at predictors of retention in patients with RA treated with a first bDMARD (second-line biologic initiation) is limited. Two studies showed that changing to a non-TNFi rather than a TNFi after the failure of a first biologic was a predictor of improved retention [31, 32]. In addition, a multivariate analysis [6] of the ACTION study showed that abatacept-treated patients who had received at least one prior biologic had a significantly lower risk of discontinuation if they were anti-cyclic citrullinated peptide (CCP) positive, had failed < 2 anti-TNF agents, or had a cardiovascular comorbidity at abatacept initiation [6]. Factors that were not predictors of abatacept discontinuation included baseline C-reactive protein value, baseline rheumatoid factor status, type of previous TNFi failure, and abatacept treatment patterns (monotherapy, combination with MTX or other csDMARDs) [6].

Our study has several strengths: we utilized a large Canadian-based registry of patients with RA, over a 9-year follow-up period, to examine the persistence with abatacept vs. a TNFi as either the first- or second-line biologic agents. To the best of our knowledge, this is the longest follow-up period examining the persistence in these patients. The results from the unselected population in this study support the clinical trial data and are more generalizable to patients found in clinical practice. Further, the Rhumadata® registry is an electronic database that contains fields that are built as an obligatory menu, limiting the possible errors and variations of data entry. This study has some limitations: as with all observational studies, there may be some bias with regard to the assignment of treatment and patient selection. Confounding by indication may be present owing to the lack of randomization. Data were collected at two academic centers and may not be generalizable to Canadian clinical practice.

## Conclusions

The results from this analysis of the Rhumadata® registry showed that abatacept and TNFis demonstrated similar persistence over a 9-year follow-up period as a first-line biologic agent in patients who have failed one prior csDMARD. As a second-line biologic agent, abatacept had a better persistence over a TNFi in patients who had failed one prior bDMARD.

## Data Availability

The datasets generated and/or analyzed during the present study are available from the corresponding author on reasonable request.

## Abbreviations

ACPA: Anti-citrullinated protein antibody
ACR: American College of Rheumatology
ACR20: American College of Rheumatology 20% improvement
AE: Adverse event
bDMARD: Biologic disease-modifying antirheumatic drug
CCI: Charlson Comorbidity Index
CCP: Cyclic citrullinated peptide
CDAI: Clinical Disease Activity Index
CI: Confidence interval
COPD: Chronic obstructive pulmonary disease
COX2: Cyclooxygenase 2
CRP: C-reactive protein
csDMARD: Conventional synthetic disease-modifying antirheumatic drug
CVD: Cardiovascular disease
DAS28-4: Disease activity score in 28 joints (four variables)
ESR: Erythrocyte sedimentation rate
HAQ: Health assessment questionnaire
HCQ: Hydroxychloroquine
HR: Hazard ratio
IR: Inadequate response
LEF: Leflunomide
MTX: Methotrexate
PGA: Physician global assessment
RA: Rheumatoid arthritis
RF: Rheumatoid factor
SAE: Serious adverse event
SC: Subcutaneous
SDAI: Simplified Disease Activity Index
SSZ: Sulfasalazine
TNFi: Tumor necrosis factor inhibitor
VAS: Visual analog scale
